# Association between obesity and periodontal disease. A systematic review of epidemiological studies and controlled clinical trials

**DOI:** 10.4317/medoral.21786

**Published:** 2017-10-21

**Authors:** Mayte Martinez-Herrera, Javier Silvestre-Rangil, Francisco-Javier Silvestre

**Affiliations:** 1 Dentist. Grant fellow (VALI+d) of the Regional Ministry Education of Valencian Community. Special Patients Unit, Department of Stomatology, University of Valencia, Valencia, Spain; 2 DDS, PhD. Associate Professor, Special Patients Unit, Department of Stomatology, University of Valencia, Valencia, Spain; 3 DDS, PhD. Assistant Professor, Special Patients Unit, Department of Stomatology, University of Valencia, Valencia, Spain; 4 MD. Service of Stomatology, University Hospital Doctor Peset, Av. Gaspar Aguilar 90, 46017 Valencia, Spain

## Abstract

**Background:**

Obesity is a very prevalent chronic disease worldwide and has been suggested to increase susceptibility of periodontitis. The aim of this paper was to provide a systematic review of the association between obesity and periodontal disease, and to determine the possible mechanisms underlying in this relationship.

**Material and Methods:**

A literature search was carried out in the databases PubMed-Medline and Embase. Controlled clinical trials and observational studies identifying periodontal and body composition parameters were selected. Each article was subjected to data extraction and quality assessment.

**Results:**

A total of 284 articles were identified, of which 64 were preselected and 28 were finally included in the review. All the studies described an association between obesity and periodontal disease, except two articles that reported no such association. Obesity is characterized by a chronic subclinical inflammation that could exacerbate other chronic inflammatory disorders like as periodontitis.

**Conclusions:**

The association between obesity and periodontitis was consistent with a compelling pattern of increased risk of periodontitis in overweight or obese individuals. Although the underlying pathophysiological mechanism remains unclear, it has been pointed out that the development of insulin resistance as a consequence of a chronic inflammatory state and oxidative stress could be implicated in the association between obesity and periodontitis. Further prospective longitudinal studies are needed to define the magnitude of this association and to elucidate the causal biological mechanisms.

** Key words:**Periodontal disease, periodontitis, periodontal infection, obesity, abdominal obesity.

## Introduction

Obesity has been described as one of the most neglected public health problems, affecting both developed and developing countries (1). The prevalence of obesity has increased substantially in the last few decades (2,3). In 2014, the World Health Organization (WHO) estimated that around 600 million obese adults worldwide were obese (4), and a further increase is expected in the future due to increased consumption of high-calorie diets and a sedentary lifestyle.

Obesity is usually defined as body mass index (BMI = kg/m2). Specifically, overweight is defined as a BMI between 25.0-29.9 kg/m2 and obesity is defined as a BMI of ≥ 30.0 kg/m2 (1-3). The exception is found in Asian regions, where overweight is defined as BMI ≥ 23 kg/m2, since in this setting obesity-related complications have been observed at comparatively lower BMI values (4). BMI is an indicator of total adiposity, but does not assess body mass distribution, so other parameters such as waist circumference (WC) or waist/hip ratio (WHR) are usually used (4). WC ≥ 102 cm in males and ≥ 88 cm in females, and WHR > 0.90 in males and > 0.85 in females indicate abdominal obesity associated to an increased risk of morbidity (1-3). These anthropometric determinations show a close correlation to the amount of visceral adipose tissue, which has been shown to be metabolically more active and secretes larger amounts of cytokines and hormones compared to subcutaneous adipose tissue (1-3).

Obesity is a chronic metabolic disease that predisposes to a variety of comorbidities including arterial hypertension, type 2 diabetes mellitus, atherosclerosis and cardiovascular diseases (3). Furthermore, obesity has been suggested to be a risk factor for periodontitis (1-3).

Periodontal disease is an infectious and inflammatory disorder of tooth-supporting structures resulting from the interaction between pathogenic bacteria and the host immune response (1). Activation of the host immune system, primarily for protective purposes, eventually leads to destruction of tissues through the synthesis and release of cytokines, proinflammatory mediators and metalloproteinases (5).

Periodontitis is among the 10 most prevalent chronic diseases affecting the world’s population (6). In recent years, research has focused on the relationship between periodontitis and systemic disorders such as diabetes mellitus, rheumatoid arthritis, cardiovascular diseases and obesity.

The association between obesity and periodontitis is one of the most recent fields of research in periodontal medicine and the possible underlying biological mechanisms remain unclear. However, adipose tissue releases proinflammatory cytokines and hormones globally referred to as adipocytokines, which induce inflammatory processes and oxidative stress disorders, generating a similar pathophysiology between the both diseases (2,3).

This association was first reported in animals in 1977 by Perlstein and Bissada, and in humans in 1998 by Saito *et al.* Since then, the hypothesis that obesity is a risk factor for periodontitis has been evidenced by several epidemiological studies (7-29).

Therefore, the aim of the present study was to offer a systematic review of the evidence on the association between obesity and periodontal disease, and of the possible mechanisms involved in this relationship. The following questions were raised in this regard: Do obese subjects have more periodontal disease? Is periodontal disease more aggressive in such population? What biological mechanisms are involved in the association between obesity and periodontal disease?

## Material and Methods

In the present study the PubMed and Embase databases were used for the collection of articles. We performed a first exploratory search through PubMed in the Medline database, using the following key words in different combinations: periodontal diseases OR periodontitis OR periodontal infection AND obesity OR abdominal obesity. The filters used in this first search were publications in English and studies conducted in humans: “Periodontal diseases”[Majr] AND “Obesity”[MeSH] AND (“humans”[MeSH Terms] AND English[lang]). Based on these keywords we obtained a total of 192 references, and the corresponding titles and abstracts were then read to compile the publications of interest.

The inclusion criteria were: controlled clinical trials, cohort and case-control studies assessing obesity and periodontal disease, studies in adults (over 18 years of age), articles published in English and after the year 2000.

Clinical cases and case series, non-human studies, studies in individuals less than 18 years of age and published in languages other than English were excluded. Furthermore, we excluded studies on metabolic syndrome and those in which the periodontal status was only assessed by tooth loss, oral hygiene, gingival appearance or use of dental prostheses. Studies that did not exclude diabetic patients or individuals with systemic inflammatory disorders, or which made no adjustment for such confounding factors, were also excluded.

A complementary search was subsequently performed in the Embase database, entering the same keywords and considering the same inclusion and exclusion criteria (‘periodontal diseases’ AND obesity AND [English]/lim AND [humans]/lim). From the articles obtained we discarded those that had already been identified in the first search in PubMed; and we obtained 2 articles that were exclusively found in Embase.

## Results - Discussion

**Figure 1:**
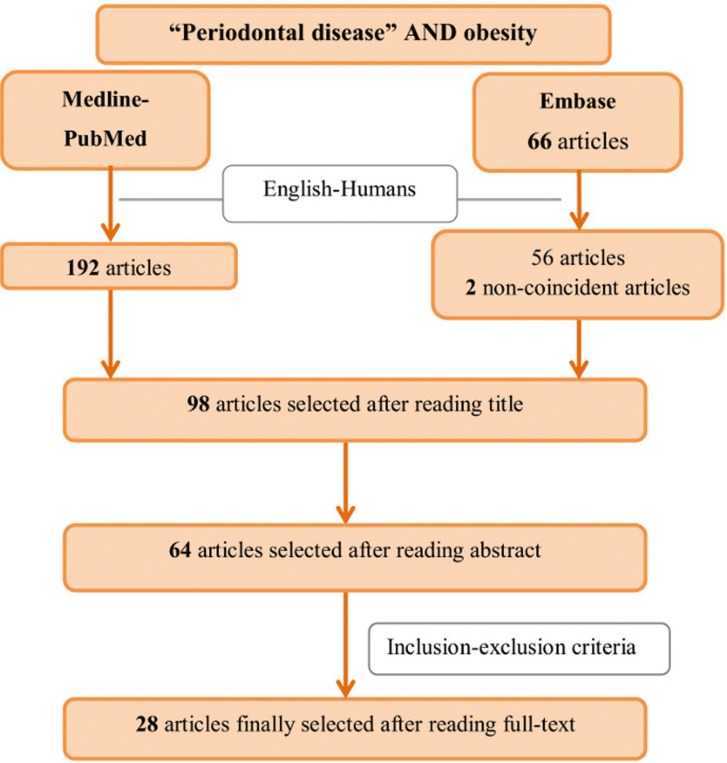
Flowchart of the search process.

The flowchart corresponding to the search process is shown in Figure 1. The search identified a total of 248 articles, of which 64 were preselected on the basis of the inclusion and exclusion criteria, and 28 were finally included in the review: 19 observational studies (7-14,16-18,20-22,24,26-29) described in  Table 1, and 9 clinical trials of periodontal treatment (30-38) reported in  Table 2. Studies are listed chronologically from the most recent article, based on the publication date (and then in alphabetical order within that same year).

**Table 1:**
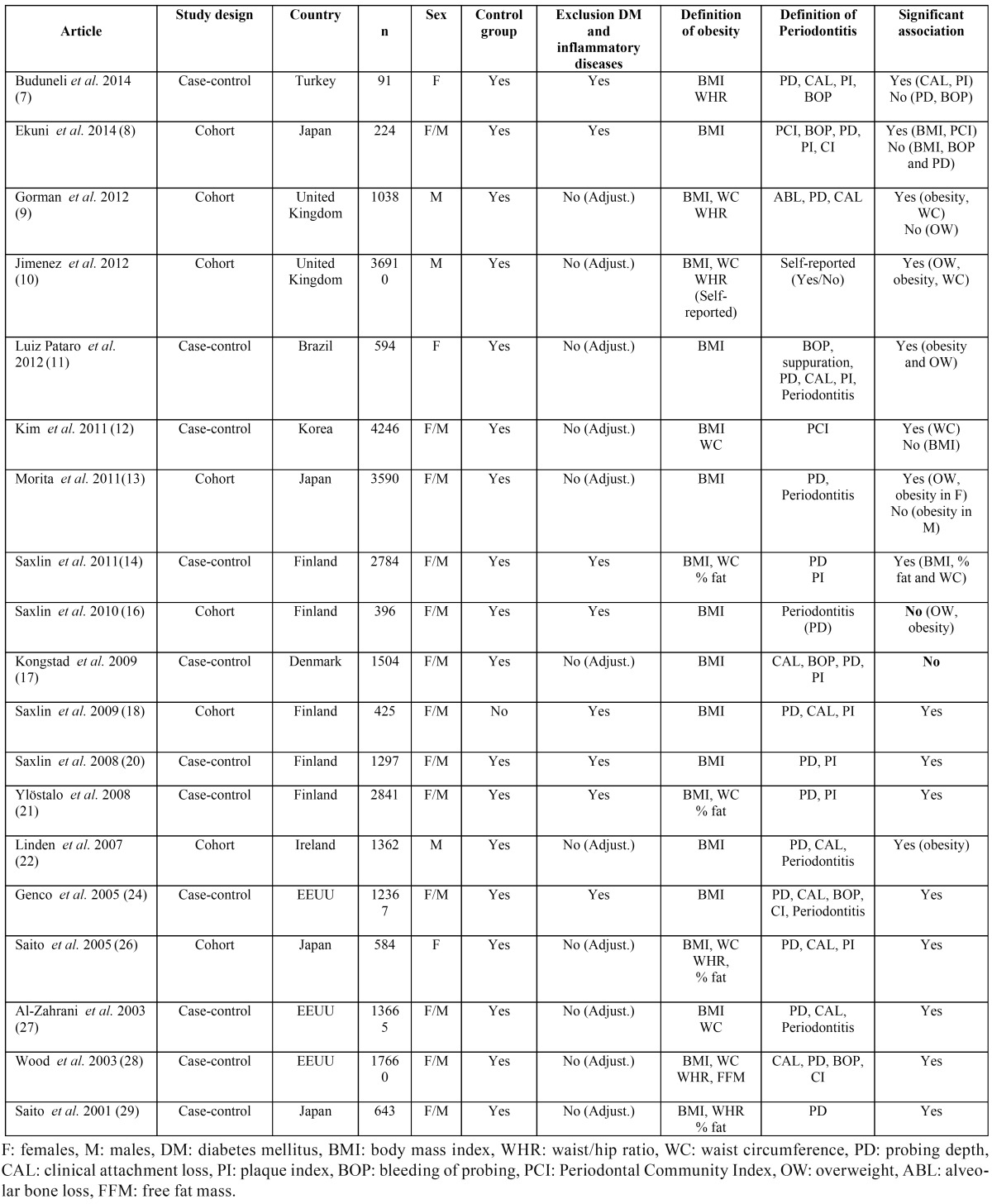
Epidemiological studies on the association between obesity and periodontal disease.

**Table 2:**
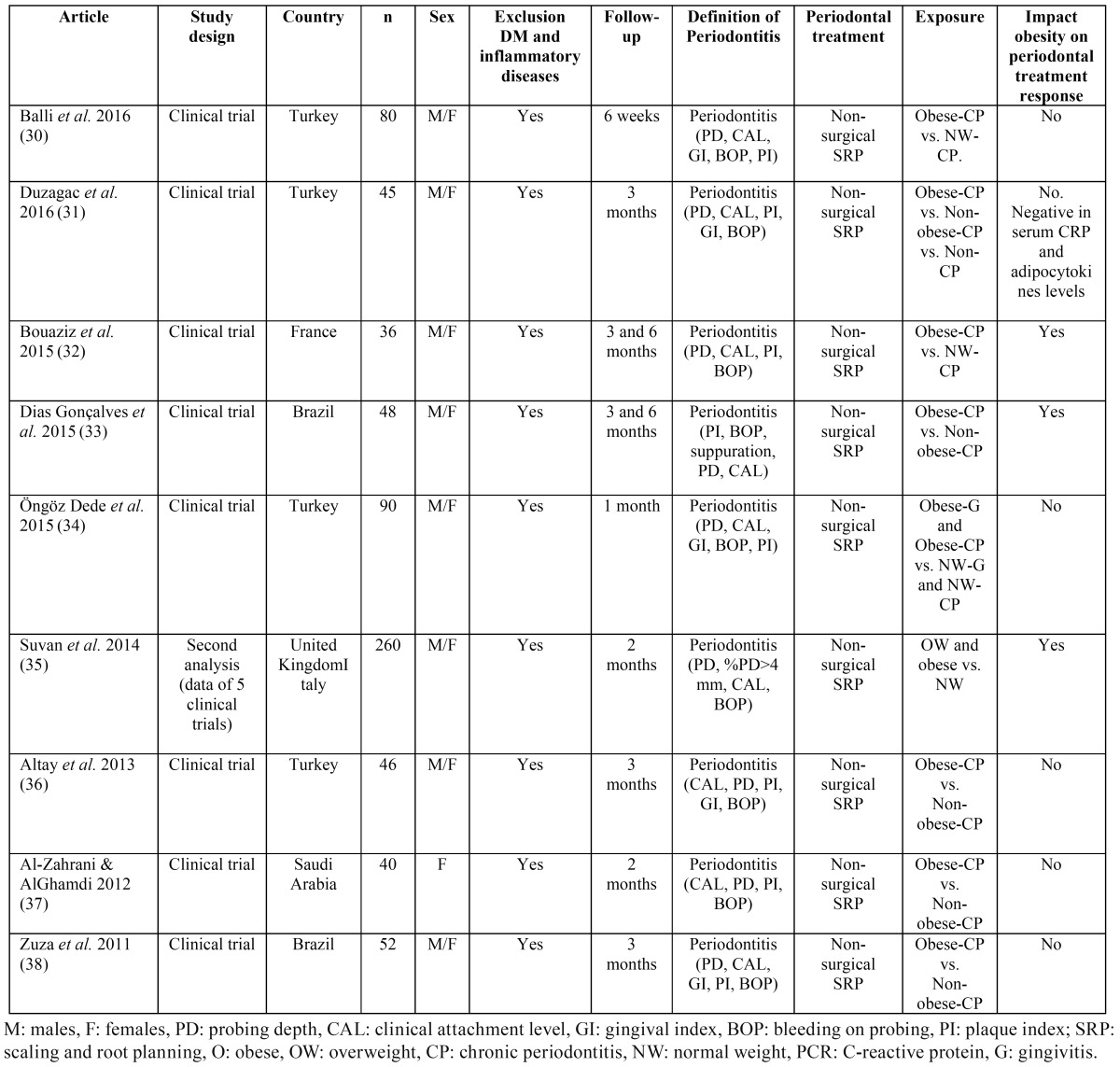
Studies of periodontal treatment in subjects with obesity and normal weight.

An association between periodontal disease and obesity has been demonstrated in the worldwide literature. Most of the 19 observational studies were of a cross-sectional nature, with very few prospective longitudinal studies (8-10,13,16,22). The results of some cross-sectional studies show that obese individuals have more periodontal disease than the normal weight population, and some publications have found this association to be stronger as the obesity level increases (11). Some of the reviewed prospective studies (8,9,13,22) suggest that overweight, obesity, weight gain and a large WC may be risk factors for the development or worsening of periodontitis. Three prospective studies (9,10,13) found a direct association between obesity and the subsequent development of periodontitis. In turn, two of these studies (10,13) also determined a direct association between overweight and the development of periodontitis.

Although most studies evidence an association between obesity and periodontal disease, of the 19 observational studies, two (16,17) described no significant association between obesity and periodontitis. Furthermore, one of them reported an inverse relationship between obesity and periodontal clinical attachment loss (17). However, these results lack a solid basis, since these studies use multiple variables using different cut-off points for evaluate obesity and periodontal disease.

Most of studies that reported an association between obesity and periodontitis focused on disease prevalence, and three informed of the extent and severity of periodontal disease (14,21,28).

In general, all the studies evaluated anthropometric and periodontal parameters to determine the association between the two disorders. All of them evaluated the degree of obesity by calculating BMI, and some of them also included other measures of abdominal obesity such as WC, WHR and, in some cases, even percentage body fat (14,21,26,28,29). A stronger correlation has been observed between periodontitis and anthropometric measures of visceral fat accumulation than between periodontitis and BMI. In fact, one of the studies reported a significant relationship between periodontal disease and WC, without correlation to BMI (12). This is consistent with the fact that abdominal adipose tissue secretes a diversity of adipocytokines, which induce inflammatory processes and oxidative stress disorders, resulting in a chronic activation of the acute phase response and the development of insulin resistance (IR).

In the same way, some studies identified a direct association between periodontitis and overweight (10,11,13), whereas in the study by Gorman *et al.* was established a correlation between periodontal disease and obesity but not overweight (9). Two longi-tudinal cohort studies (8,9) determined that weight gain was directly associated to the development of periodontitis. In contrast, the study by Linden *et al.* found no association between weight gain from 21 to 60-70 years of age and the severity of periodontitis, determining that a high BMI at an early age is not predictive of periodontitis in old age (22).

The evaluation of periodontal disease was seen to vary greatly in the research setting. Some studies evaluated and compared periodontal parameters such as probing depth (PD), clinical attachment level (CAL), bleeding on probing (BOP), gingival index (GI), or plaque index (PI) and calculus index (CI). The larger studies (8,12) used the community periodontal index (CPI) and restricted measurements to representative teeth or quadrants. In turn, some studies established a diagnosis of periodontitis and classified the population according to the presence or absence of periodontitis, determining prevalence. The greatest agreement among the different studies was referred to the definition of periodontal disease as PD ≥ 4 mm and CAL ≥ 3 mm, although different definitions and combinations were employed. The nine interventional studies used the different periodontal parameters (PD, CAL, BOP, PI, GI) to assess periodontal disease and the changes following periodontal treatment.

Of the 28 articles reviewed, three included only males (9,10,22), four included only females (7,11,26,37), and the remainder included individuals of both sexes. Of the latter, three studies (13,14,17) analyzed males and females separately, and another 10 articles adjusted the analysis by gender (8,12,16,18,20,21,24,27-29). Some studies (15,24) indicate that males are at a greater risk of developing periodontitis than females, while other articles (13,39) suggest that females may be more vulnerable to periodontitis, due to hormonal fluctuations that increase gingival inflammation. Nevertheless, Saxlin *et al.* (14,16) suggest that gender does not appear to influence the association between obesity and periodontitis. Consequently, on the basis of the results of our review, it is not possible to draw firm conclusions regarding the influence of gender upon the association between obesity and the development of periodontitis.

Age has also been considered as a risk factor for periodontitis and has been observed that the prevalence and severity of periodontitis increase with age, probably as a consequence of the longer exposure of the periodontal tissues to bacterial plaque (39). However, since most of the included studies (8,9-14,16-18,20-22,24,26-29) adjusted the results according to age, the association between obesity and periodontitis seems to be independent of age. Regard to smoking habit, eleven studies (8,16,18,20,30-34,37,38) excluded smokers, twelve adjusted the analysis for smoking as a confounding factor (9-13,22,24,26-29,35), and three studies divided the study population into smokers and non-smokers (7,14,17). In one of the latter, non-smoking obese women had a greater clinical attachment loss and greater bleeding and plaque index than non-obese and non-smoking women (7). Likewise, another two studies reported an association between the number of teeth with pathological periodontal pocket depths greater than or equal to 4 mm and BMI in a non-smoking subpopulation (14,21). Therefore, although smoking habit predisposes to periodontitis and contributes to periodontal tissue destruction (39), it has been observed that smoking and obesity are independent risk indicators for periodontitis, and both conditions show a dose-dependent correlation to the risk of periodontitis (25).

This review has focused on studies that only examine the association between obesity and periodontitis, without considering other systemic diseases or oral disorders. As well as, we considered whether the study included a control group for comparison versus the obese individuals in order to establish a genuine association between obesity and periodontal disease. In this way, we considered whether the studies controlled for confounding factors by excluding those subjects with diabetes mellitus or other concomitant inflammatory conditions capable of influencing the results. Of the 28 articles reviewed, 17 excluded diabetic patients (7,8,14,16,18,20,21,24,30-38) and 11 controlled for diabetes as a confounding factor (9-13,17,22,26-29). Nevertheless, it must be taken into account that despite the control of this confounding factor, those studies that did not exclude diabetic individuals were susceptible to possible bias in the interpretation of the results obtained.

Of the nine studies evaluating the effect of obesity upon the clinical and biochemical response to periodontal treatment, six (30,31,34,36-38) concluded that obesity does not exert a negative modifying effect upon the outcome of non-surgical periodontal treatment. In contrast, three articles (32,33,35) suggested that obesity exerts a negative effect upon periodontal treatment response. Therefore, to date, the effect of obesity on the response to periodontal treatment remains uncertain.

Some of the reviewed studies analyzed proinflammatory molecules in serum, in an attempt to identify a possible causal mechanism relating obesity to periodontitis. Most of the studies pointed to the inflammatory process as the possible cause. In obesity, adipocytes secrete proinflammatory cytokines such as TNF-α and IL-6, which stimulate the hepatic production of acute phase reactants such as C-reactive protein (CRP) and cause alterations in the host’s immune response – increasing the susceptibility to bacterial infection (6). Likewise, TNF-α is one of the first proinflammatory cytokines induced by the pathogens of periodontitis. TNF-α contributes to the onset of periodontitis through the stimulation of osteoclast formation, inducing alveolar bone destruction and connective tissue degradation (40). It is therefore believed that TNF-α mainly contributes to the early stages in the development of periodontal disease in obese individuals, not to the worsening and/or progression of already established periodontitis. It has been reported that the serum TNF-α levels were not correlated to the severity of destructive periodontal disease in patients with BMI > 30 kg/m2 (24).

Respect to IL-6, although in one study higher serum interleukin-6 (IL-6) levels were reported in obese individuals (7) and Saxlin *et al.* (18) found the serum IL-6 levels to be associated to periodontal infection, the association between IL-6 and periodontitis remains unclear, due to its dual pro- and antiinflammatory effects (40).

Similarly, the role of other molecules in the association between obesity and periodontitis, such as leptin, adiponectin and resistin, remain to be clarified. Leptin plays a protective role in immune system function, and is present in larger amounts in minimally inflamed gingival tissue, decreasing its concentration at sites presenting deeper pocket depths (3). Serum adiponectin - which exerts antiinflammatory effects - tends to decrease, and resistin - which exerts inflammatory effects - tends to increase in individuals with periodontitis (19).

Likewise, it has been reported that obesity may be associated to periodontitis through the increased production of reactive oxygen species (ROS). Excessive ROS levels and a decrease in antioxidant substances in the periodontal tissues result in a chronic activation of inflammation and tissue destruction (40). This chronic inflammation and oxidative stress could condition the development of IR. Recently, it has been suggested that IR plays a role in the pathogenesis of periodontitis (24). It has been seen that non-surgical periodontal treatment results in lowered serum TNF-α levels and decreased IR among the obese population (31,36).

**Figure 2:**
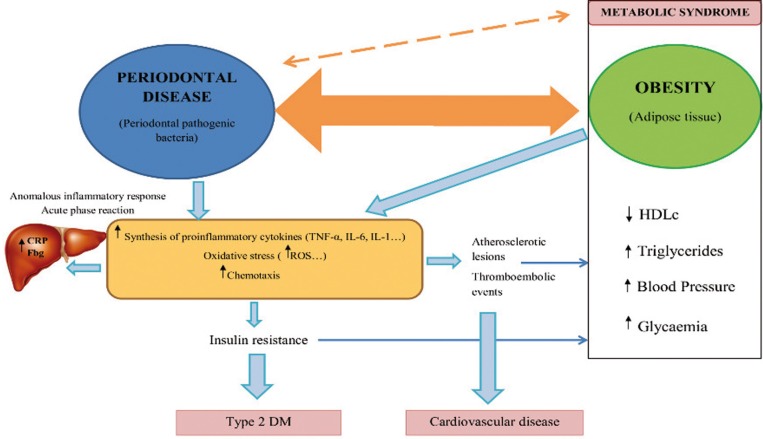
Model of association between obesity and periodontal disease through inflammation, oxidative stress and insulin resistance.Abbreviations: CRP: C-reactive protein, Fbg: fibrinogen, TNF-α: tumor necrosis factor alpha, IL-6: inteleukin 6, IL-1: interleukin 1, ROS: reactive oxygen species, DM: diabetes mellitus, HDLc: HDL cholesterol.

Figure 2 shows a model of association between obesity and periodontal disease through inflammation and oxidative stress. However, the pathophysiological mechanism whereby obesity affects the periodontium remains unclear, and this association may be bidirectional (3,40).

Most of the included studies involved a cross-sectional design, thereby precluding definition of the mechanisms underlying the association between both diseases. Further prospective studies are needed to confirm the causal relationship between obesity and periodontitis, as well as the pathophysiological mechanism involved in the association between both diseases.

## Conclusions

The results of this study indicate the existence of an association between obesity and periodontitis and although the causal mechanisms underlying this association remain unclear, the development of insulin resistance as a consequence of a chronic inflammatory state and oxidative stress could be implicated in the association between obesity and periodontitis. This evidence points to the need for further prospective clinical studies, designed to define the magnitude of this association and clarify the role of IR in the pathogenesis of periodontitis.
